# M1 transcranial direct current stimulation augments laparoscopic surgical skill acquisition

**DOI:** 10.1038/s41598-023-40440-x

**Published:** 2023-08-23

**Authors:** Daniel Galvin, Adam J. Toth, Barry O’Reilly, Ray O’Sullivan, Mark J. Campbell

**Affiliations:** 1 ASSERT Centre, College of Medicine and Health, University College Cork, Cork, Ireland; 2 Lero, The Science Foundation Ireland Research Centre for Software, University of Limerick, Limerick, Ireland; 3 Department of Physical Education and Sport Sciences, University of Limerick, Limerick, Ireland

**Keywords:** Motor control, Motor cortex, Synaptic plasticity

## Abstract

The acquisition of basic surgical skills is a key component of medical education and trainees in laparoscopic surgery typically begin developing their skills using simulation box trainers. However, despite the advantages of simulation surgical training, access can be difficult for many trainees. One technique that has shown promise to enhance the deliberate practice of motor skills is transcranial electric stimulation (tES). The purpose of this study was to assess the impact of transcranial direct current stimulation (tDCS) on training induced improvements and retention of traditional time and kinematic based laparoscopic surgical skill metrics. Forty-nine medical students were randomly allocated to a neurostimulation or sham group and completed 5 training sessions of a bead transfer and threading laparoscopic task. Participants in both the sham and stimulation groups significantly improved their time and kinematic performance on both tasks following training. Although we did find that participants who received M1 tDCS saw greater performance benefits in response to training on a bead transfer task compared to those receiving sham stimulation no effect of neurostimulation was found for the threading task. This finding raises new questions regarding the effect that motor task complexity has on the efficacy of neurostimulation to augment training induced improvement and contributes to a growing body of research investigating the effects of neurostimulation on the sensory-motor performance of laparoscopic surgical skill.

## Introduction

The acquisition of basic surgical skills is a key component of medical education for medical students and junior surgical trainees. Laparoscopic surgery is a minimally invasive surgical technique that involves the introduction of trocars and instruments through small incisions in the patient’s abdomen^1^. The operative field is visualised using a camera inserted through the patient’s umbilicus. Laparoscopy has many advantages over traditional open surgery particularly in terms of recovery time, blood loss and pain. Therefore, laparoscopy has been adopted widely in many surgical subspecialities^2^ and trainee surgeons must now acquire laparoscopic surgical skills as part of surgical training programs^3^.

Trainees in laparoscopic surgery typically begin developing their skills using simulation box trainers. Simulation of basic surgical tasks provides a safe platform for surgical trainees to develop the requisite cognitive and motor skills with no risk to patient outcomes. A variety of tasks can be administered and specific programs such as the Fundamentals of Laparoscopic Surgery (FLS) have been developed and incorporated into surgical training curricula worldwide^4^. Despite the advantages of simulation surgical training, access to training can be difficult for many trainees. Clinical workloads^5^, the availability of simulation facilities^5^ and competing demands for limited resources^6^ have been highlighted as obstacles hindering the ability for trainees to access simulated surgical training. Recently, the coronavirus pandemic has further impacted on the ability to provide training and accommodate the volume of trainees^7^. This limited exposure to training resources has the potential to hinder surgical performance and patient health^8,9^*.* As such, there is an increasing appetite in medicine to explore methods that may either increase both access to and the effectiveness of simulated surgical motor skill training on surgical performance.

In recent years, one technique that has shown promise as an adjunct to the deliberate practice of motor skill is transcranial electric stimulation (tES). Typically administered as a direct current (tDCS), this form of neurostimulation involves passing small electrical currents (1–3 mA) over the scalp to modulate the excitability of underlying cortical neurons for up to 60 min following stimulation^10–12^. When applied over the relevant limb representation of the motor cortex, tDCS has shown to accelerate improvements of various motor skills following training, including musical skills^13,14^ and sport skills^15,16^. As a result of the promise of this technique, commercially available tDCS devices have become available and received both FDA and CE approval. One such device is the Halo Neuroscience tDCS device (FlowNeuroscience™) which has been recently shown to accelerate training induced performance improvements of motor skill in video gaming tasks^17^.

Over the past 5 years, emerging research has been conducted examining the impact of tDCS on surgical skill acquisition. The few studies to date vary not only according to their methodological rigour but also by the site of neurostimulation, the tasks experimenters evaluated and the metrics that were used to quantify performance. For example, stimulation has largely been administered over the prefrontal cortex^18,19^, primary motor cortex^20–23^ or supplementary motor area (SMA^21^;), with most manually evaluating basic metrics of performance including the time to complete the task^19,24^ or a score related to the number of errors made^18,20,21,25^. However, research highlighting the need for more robust and objective metrics to quantify surgical performance has shown that the inclusion of biomechanical measures such as instrument movement efficiency, velocity, and positioning can provide a more complete description of surgical skill^26–28^.

One such system that incorporates software that can integrate with traditional simulation box trainers to provide surgical trainees with biomechanical kinematic data of the implements during simulated surgical tasks has been developed by the company, LAPARO (Laparo analytic, Laparo Medical Simulators, Poland). Since its inception in 2015, this system has now been integrated in medical settings in over 90 countries around the world in over 1000 hospitals, medical schools, and simulation centers. Despite its widespread acceptance, no study to date has assessed the effect of tDCS on laparoscopic skill acquisition incorporating performance metrics from the LAPARO software. By determining the kinematic markers of surgical skill improvement and the effect that tDCS can have on these markers across training, we can better understand how to evaluate surgical skill and coach surgical trainees.

The purpose of this study was to assess the impact that tDCS may have on the training induced improvement and retention of traditional time and kinematic based laparoscopic surgical skill metrics. We hypothesised that those receiving tDCS applied over the hand regions of M1 would show greater training-mediated improvements in the time required to complete simulated surgical tasks, in the distance implements travelled over the course of the task, in the average velocity of the implements across tasks and through a reduction in the number of excessive velocity events, compared to those receiving a sham stimulation.

## Results

Fifty-three (N = 53; 27 Female) young healthy adult participants aged 23.43 ± 2.96 years (Mean ± SD) were recruited from the University College Cork medical student population. All participants were naïve to laparoscopic skills training. After exclusions (see Methods below) 49 participants (N = 49; 22 Male) were brought forward for data processing and analysis. Independent sample t-tests revealed no significant differences between male and female participants for any performance metric in both the bead transfer and threading tasks (Supplementary File 1).

### Time to complete (TTC)

**Figure 1:**
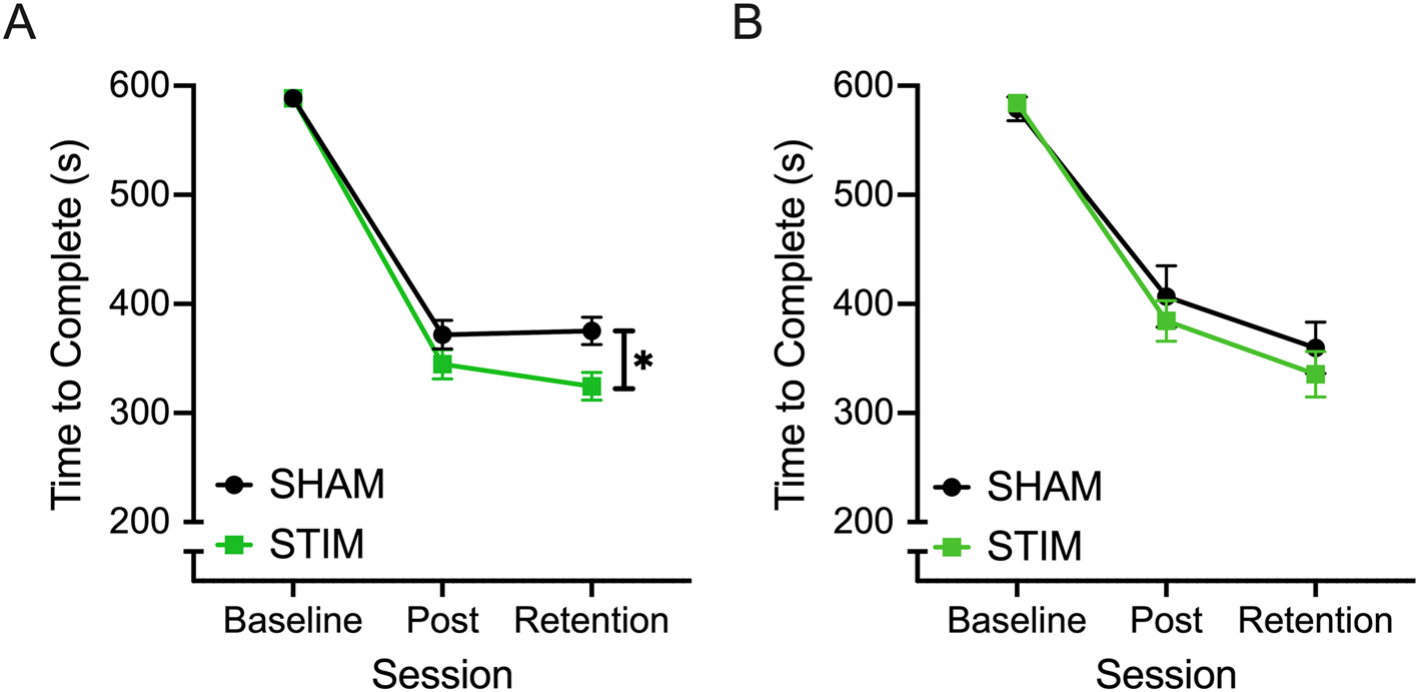
The average (± SE) time (in seconds) required to complete the bead transfer (**A**) and threading (**B**) tasks at Baseline, Post and Retention sessions by participants who received active tDCS (STIM; green) and sham tDCS (SHAM; black). *Represents significant differences between neurostimulation groups at an alpha level of 0.05.

The effect of session (F_(1.608, 107.724)_ = 525.407, *p* < 0.001, ƞ^2^ = 0.887) and the interaction effect between session and stimulation condition (F_(1.608, 107.724)_ = 5.788, *p* = 0.007, ƞ^2^ = 0.080) were both significant for TTC in the bead transfer task. Post hoc comparisons revealed that participants in both stimulation conditions significantly reduced the time required to complete the bead transfer task after 5 days of training (*p* < 0.001) and that this reduction was maintained at the retention test 5 days after the Post test (Post vs. Retention: Stim; *p* = 0.063, Sham; *p* = 0.972). After confirming no significant difference in baseline TTC performance between stimulation conditions, ANCOVA analysis revealed that after controlling for variations in baseline performance, a significant main effect of condition was found (F_(1, 66)_ = 5.028, *p* = 0.028, ƞ^2^ = 0.071) where those in the stim group were able to reduce their bead transfer completion time by 38.989 ± 17.389 s more than those in the sham group (Fig. 1A).

In the threading task, a significant main effect of session was found (F_(2, 168)_ = 280.318, *p* < 0.001, ƞ^2^ = 0.769) for TTC in the threading task (Fig. 1B). Post hoc comparisons revealed that all participants were able to improve their performance by 185.804 ± 10.791 s between Baseline and Post sessions and by a further 48.030 ± 9.347 s between Post and Retention sessions (all *p* < 0.001). No main effect was found for stimulation condition or implement, nor were any significant interaction effects found (Supplementary File 1).

### Distance travelled

**Figure 2:**
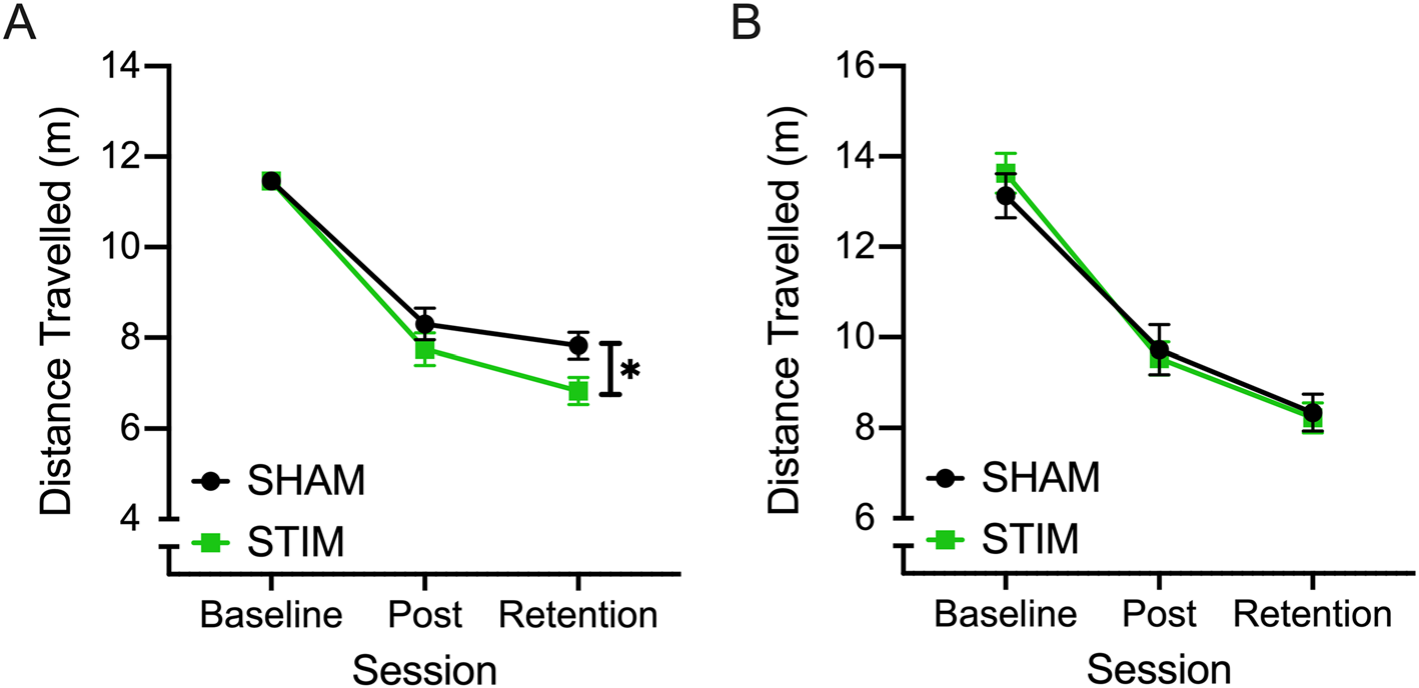
The average (± SE) distance travelled (in metres) by both implements during completion of the bead transfer (**A**) and threading (**B**) tasks at Baseline, Post and Retention sessions by participants who received active tDCS (STIM; green) and sham tDCS (SHAM; black). *Represents significant differences between neurostimulation groups at an alpha level of 0.05.

For Distance Travelled, both the main effect of session (F_(1.439, 96.401)_ = 191.134, *p* < 0.001, ƞ^2^ = 0.740) and interaction between session and stimulation condition were significant, (F_(1.439, 96.401)_ = 4.704, *p* = 0.020, ƞ^2^ = 0.066). Post hoc comparisons revealed that participants in both stimulation conditions significantly reduced the distance their implements travelled to complete the bead transfer task after 5 days of training (*p* < 0.001). Moreover, while this reduction was maintained at the retention test 5 days after the Post test for the sham group (Post vs. Retention: Sham; *p* = 0.081), further improvements were observed at retention for the stim group (Post vs. Retention: Stim; *p* < 0.001). After confirming no significant difference in baseline TTC performance between stimulation conditions, ANCOVA analysis revealed that after controlling for variations in baseline performance, a significant main effect of implement was found (F_(1, 66)_ = 9.326, *p* = 0.003, ƞ^2^ = 0.124). While the effect of condition was not significant (F_(1, 66)_ = 3.088, *p* = 0.084, ƞ^2^ = 0.045), there was a trend for participants in the stim group to reduce the amount their instruments travelled for in Post and Retention sessions by 77.449 cm more than those in the sham group (Fig. 2A).

In the threading task, a significant main effect of session was found (F_(2, 166)_ = 122.697, *p* < 0.001, ƞ^2^ = 0.596) for TTC in the threading task. Post hoc comparisons revealed that participants were able to reduce the amount their implements travelled during the task by 3.751 ± 0.357 m between Baseline and Post sessions and by a further 1.347 ± 0.268 m between Post and Retention sessions (all *p* < 0.001). No main effect was found for stimulation condition or implement, nor were any significant interaction effects found (Fig. 2B) (Supplementary File 1).

### Velocity

**Figure 3:**
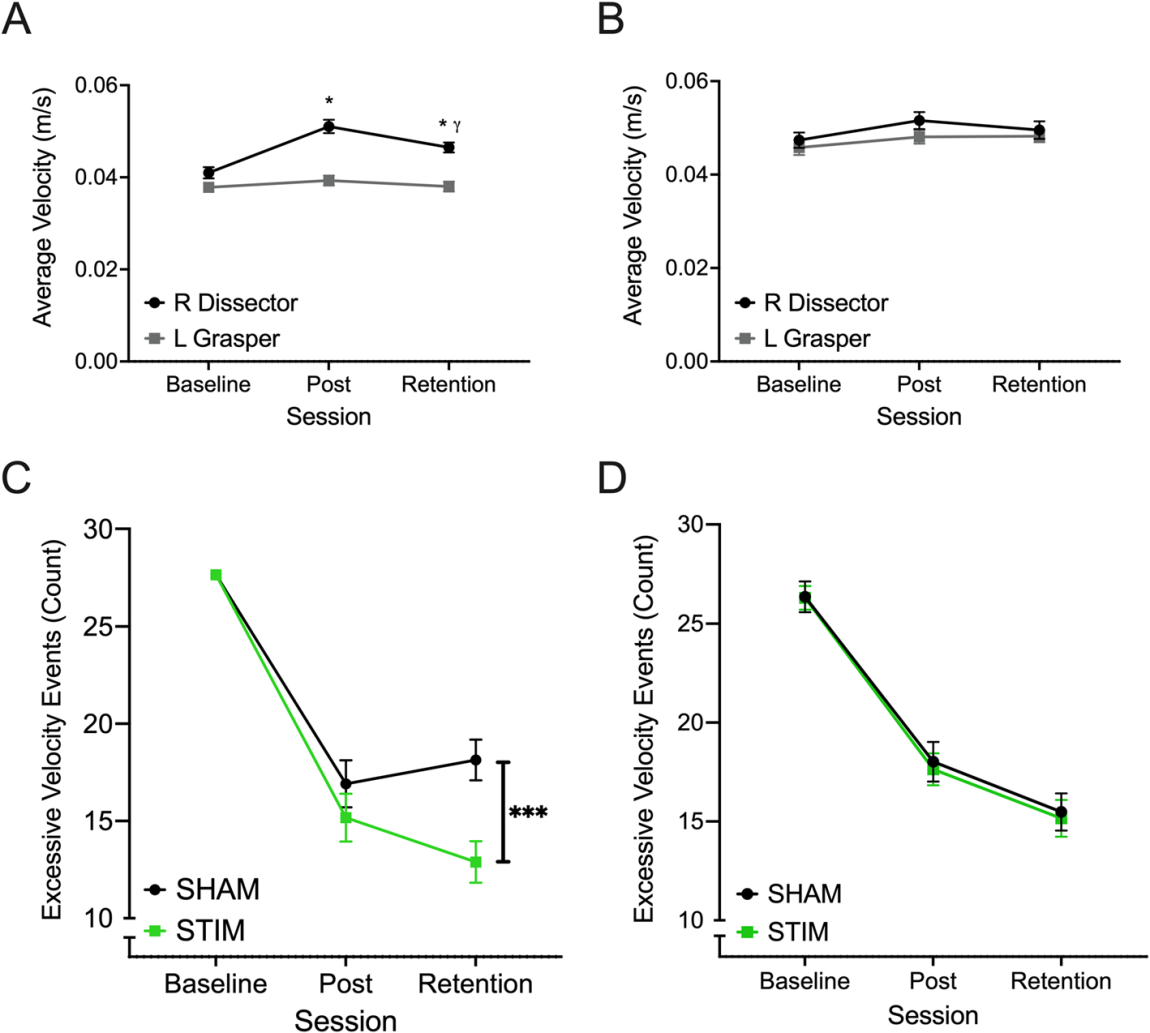
A & B: The average (± SE) velocity of the left (grey) and right (black) implements during completion of the bead transfer (**A**) and threading (**B**) tasks across Baseline, Post and Retention sessions. *And γ represent significant differences from Baseline and Post sessions respectively at an alpha level of 0.05. C & D: The average (± SE) number of excessive velocity events by both implements during completion of the bead transfer (**C**) and threading (**D**) tasks at Baseline, Post and Retention sessions by participants who received active tDCS (STIM) and sham tDCS (SHAM). ***Represents significant differences between neurostimulation groups at an alpha level of 0.001.

When considering the average velocity of the implements during the bead transfer task, the effect of session (F_(1.582, 106.026)_ = 29.716, *p* < 0.001, ƞ^2^ = 0.887), implement (F_(1, 67)_ = 33.101, *p* < 0.001, ƞ^2^ = 0.331) and the interaction between these two variables (F_(1.582, 106.026)_ = 16.509, *p* < 0.001, ƞ^2^ = 0.198) were all significant. Post hoc comparisons revealed that while the average velocity of the Grasper in the left hand did not significantly differ across sessions (all *p* > 0.126), the velocity of the Dissector, in the right hand, significantly increased between Baseline and Post sessions (*p* < 0.001) and then decreased between Post and Retention sessions (*p* < 0.001) while remaining higher than the velocity at baseline (*p* < 0.001) (Fig. 3A). No effect of stimulation condition was found for velocity during the bead transfer task.

In the threading task, a significant effect of session was found (F_(1.734, 143.962)_ = 6.083, *p* = 0.003, ƞ^2^ = 0.068) with post hoc comparisons revealing that the average speed of participants’ implements was significantly higher during the Post session compared to the Baseline (*p* = 0.007) but not Retention (*p* = 0.482) sessions (Fig. 3B).

### Excessive velocity events

In the bead transfer task, main effects of implement (F_(1, 67)_ = 4.449, *p* = 0.039, ƞ^2^ = 0.062), session (F_(2, 134)_ = 94.522, *p* < 0.001, ƞ^2^ = 0.585), and condition (F_(1, 67)_ = 4.618, *p* = 0.035, ƞ^2^ = 0.064) were all significant, as was the interaction between session and stimulation condition (F_(2, 134)_ = 4.604, *p* = 0.012, ƞ^2^ = 0.064). Post hoc analyses revealed that in the Retention session, the stimulation group had significantly fewer excessive velocity events compared to the sham group. After confirming no significant difference in baseline TTC performance between stimulation conditions, ANCOVA analysis revealed that after controlling for variations in baseline performance, a significant main effect of condition was found (F_(1, 66)_ = 6.848, *p* = 0.011, ƞ^2^ = 0.094) where those in the stim group were able to significantly reduce the number of excessive velocity events by 3.490 ± 1.334 s more than those in the sham group (Fig. 3C).

In the threading task, significant main effects of session (F_(2, 166)_ = 123.218, *p* < 0.001, ƞ^2^ = 0.598) and implement (F_(1, 83)_ = 4.791, *p* = 0.031, ƞ^2^ = 0.055) were found. Post hoc comparisons showed that 1.851 ± 0.846 fewer excessive velocity events were detected for the dissector in the right hand compared to the grasper in the left hand. Moreover, participants were able to significantly reduce the number of excessive velocity events during task completion across all 3 testing sessions (all *p* = 0.001) (Fig. 3D).

## Discussion

The purpose of this study was to assess the impact of tDCS on the training induced improvement and retention of laparoscopic surgical skill. Two commonly used tasks were assessed, a bead transfer, and a threading task. Participants in both the sham and stimulation groups significantly improved their performance on both tasks following a training protocol that involved 5 sessions each 20 min in duration (10 min per task). While no significant difference in the level of improvement on the threading task between participants in the sham and stimulation groups was found, we did find that those participants who received bipolar bihemispheric tDCS applied over the hand regions of M1 improved both their time and kinematic based performance metrics on the bead transfer task to a greater extent than those who trained whilst receiving a sham stimulation. Furthermore, this enhanced improvement observed among the stimulation group was maintained 5 days later at the retention test. We discuss these findings within the context of training induced sensory-motor skill improvements, the effect of tDCS on the enhancement of motor skill and the future implications of enhancing surgical skill through neuromodulation techniques on the medical profession.

In this study, novice surgical participants in both stimulation conditions significantly reduced the time required to complete the bead transfer task and this reduction was maintained at the retention test 5 days following completion of the Post test. However, the group receiving neurostimulation demonstrated a 5% improvement in time to completion compared to the sham group. This 5% equates to a 30 s faster time to complete the bead transfer task compared to the sham group at the retention time point. This finding corroborates the work by Cox and colleagues^21^ who showed the added improvement of tDCS over the SMA on a surgical peg transfer task to be 3% when compared to sham stimulation. Furthermore, Friehs and colleagues^29^ reported a 4.5% improvement on a digital stop-signal game measuring response inhibition.

On the threading task both groups significantly improved over the course of training from pre to post/retention but there was no significant effect of neurostimulation. The stim group did, on average however, complete the task 17 s quicker than the sham group. Turning to the findings for implements distance travelled, participants in both stimulation conditions significantly reduced the distance their implements travelled to complete the bead transfer task after 5 days of training. Moreover, while this reduction was maintained at the retention test 5 days after the Post test for the sham group, further improvements were observed at retention for the stim group. A smaller distance of travel by the implements would indicate a safer more efficient laparoscopic procedure. Other surgical skills research reports a decrease in error scores coupled with quicker completion times (see^19,21^). In the threading task, participants were able to reduce the amount their implements travelled during the task between Baseline and Post sessions and between Post and Retention sessions but the stim and sham groups did not significantly differ.

Finally, we sought to examine the steadiness or smoothness with which participants controlled the implements during the completion of both tasks and were able to do so by measuring implement velocity and defining excessive velocity events. In the bead transfer task, main effects of implement, session, and condition were all significant, as was the interaction between session and stimulation condition. Post hoc analyses revealed that in the Retention session, the stimulation group had significantly fewer excessive velocity events compared to the sham group. After confirming no significant difference in baseline TTC performance between stimulation conditions, we showed that after controlling for variations in baseline performance, a significant main effect of neurostimulation was found where those who received tDCS were able to significantly reduce the number of excessive velocity events more than those in the sham group over the course of training (Fig. 3C). In the threading task, significant main effects of session and implement were found but again the effect of neurostimulation was not significant (Fig. 3D).

The observed improvements by the stimulation group over and above those receiving sham stimulation equate to a significant time improvement, coupled with reduced instrument travel distance and improved implement control (increase in movement smoothness observed as a reduction in EVEs). These reductions are similar to training induced improvements in other sensory-motor and digital tasks^17,29^ as well as surgical training (^20,21^; Peg Transfer task^30^; Pattern Cutting). For example, Toth noted a 6.1–8.9% improvement for the M1 tDCS stim group (high skill/low skill groups) over a sham group for a target acquisition task over the same time course of training (5 days × 10 min per day training × 20 min of stim/sham).

However, while the beads task was able to successfully demonstrate a significant positive effect of bipolar bihemspheric M1 stimulation, the threading task did not. For now, we hypothesize that it may be that the beads task is a highly repetitive highly consistent spatio-temporal task that may be more conducive to the effects of brain stimulation during training. The threading task is more complex and variable from a sensorimotor standpoint where the movement and positioning of the implements appear to differ more greatly across multiple attempts at completing the task. Previous work has shown that as task complexity increases, brain activation extends towards prefrontal, parietal and temporal areas^31,32^. As a result, neurostimulation of the bilateral M1 hand regions may not be sufficient to modulate more complex task performance over the time-period observed in this study. This hypothesis aligns with existing work showing a lack of an effect of tDCS during more complex tasks^33^. Overall, future work may look to characterising the variability or complexity of various surgical tasks to better establish optimal time-periods over which the effects of tDCS may manifest, or also explore to a greater extent the efficacy of multisite neurostimulation protocols on exacerbating the benefits of neurostimulation to the development and maintenance of surgical expertise.

The findings and implications from this research may be applied to several research areas and real-world situations. For example, when learning a new task, we replicate that performance can vary considerably in novice performers^34^. Awareness of this knowledge may provide encouragement and facilitate resilience in novice performers who may be prone to frustration and decreased motivation due to their initially high performance variability when learning a new skill. This knowledge also can be applied into existing self-regulated learning models of deliberate practice^35^. Secondly, since more time and effort are required to manifest appreciable increases in performance as expertise increases, cognitive strategies such as mental practice (MP)^36^ and action observation (AO)^37^ may be implemented given their previously shown effectiveness for augmenting performance. The use of motor simulation strategies like MP and AO may be especially relevant in surgical skills training, where the effect of these cognitive strategies on performance has yet to be investigated with any rigour. Thirdly, our work has corroborated previous research in showing that tDCS is highly efficacious for skill acquisition among novice performers^34^. Overall, tDCS is a promising technique in both performance and clinical contexts and we encourage future research to continue to explore its merit.

## Conclusion

Overall, this study demonstrates that magnitude of performance improvement on two laparoscopic training skills, bead transfer and threading, among novice surgical trainees over the course of 1 week of training (100 min). Secondly, we showed that transcranial direct current stimulation (tDCS) can accelerate motor performance improvements, and that the effect of tDCS was potentially confined to the bead transfer task over the training period examined due to its simplicity compared to the threading task. This work significantly contributes to a growing body of research investigating the effects of neurostimulation on sensory-motor performance and demonstrates laparoscopic simulation training as a fruitful avenue to study motor learning and the effects of neurostimulation on motor skill development.

## Methods

### Participants

Each participant was provided with a study information leaflet and provided their informed written consent prior to participating in the study. The experiment was approved by the clinical research ethics committee of the Cork Teaching Hospitals at University College Cork as well as the University of Limerick research ethics board in accordance with the Declaration of Helsinki.

### Testing and training environment

All testing and training sessions were conducted at the ASSERT surgical simulation lab at University College Cork. A laparoscopic skill analyser was used to assess performance on surgical tasks at baseline, post-training and retention sessions (Laparo analytic, Laparo Medical Simulators, Poland). Three box trainers (identical to that used at the laparoscopic analyser station; Laparo Aspire, Laparo Medical Simulators, Poland) were setup in one area of the room for training sessions, while in another area of the room, 2 stations were allocated to the neurostimulation component of the experimental protocol.

### Questionnaires

Upon entering the simulation lab on Day 1, participants completed a questionnaire that captured information regarding their age, sex and the number of hours they reported video gaming on average per week. Their handedness was assessed using the Edinburgh Handedness Scale. Participants were excluded if they reported any diagnosed neurological or neuromuscular disorder or if they were left-handed (1 excluded). To mitigate any confounding effects of caffeine or other neuroactive substances, participants were asked whether they were taking any medications at the time of testing and to refrain from caffeine and alcohol within 4–6 h^38^ of attending each daily experimental session and an experimenter recorded whether or not they had consumed any caffeine upon arriving to the lab each day. Future research may more explicitly control for the use of specific neuroactive substance use that may affect motor learning outcomes.

On the first and final training days participants also completed the 32-item Brunel Mood State (BRUMs) questionnaire^39^ and the Pittsburgh Sleep Quality Index (PSQI)^40^ to account for any effects on performance due to changes in mood or sleep quality respectively. The BRUMs questionnaire comprises 32 different mood descriptors and participants were asked to indicate the extent to which each descriptor matched their current mood on a Likert scale from 1 to 4. The PSQI is a questionnaire that poses questions regarding a participant’s sleep over the previous month. The questions in the PSQI were modified for participants on their final training day to inquire about their sleep over the previous week while enrolled in the study.

### Baseline, post and retention testing

**Figure 4:**
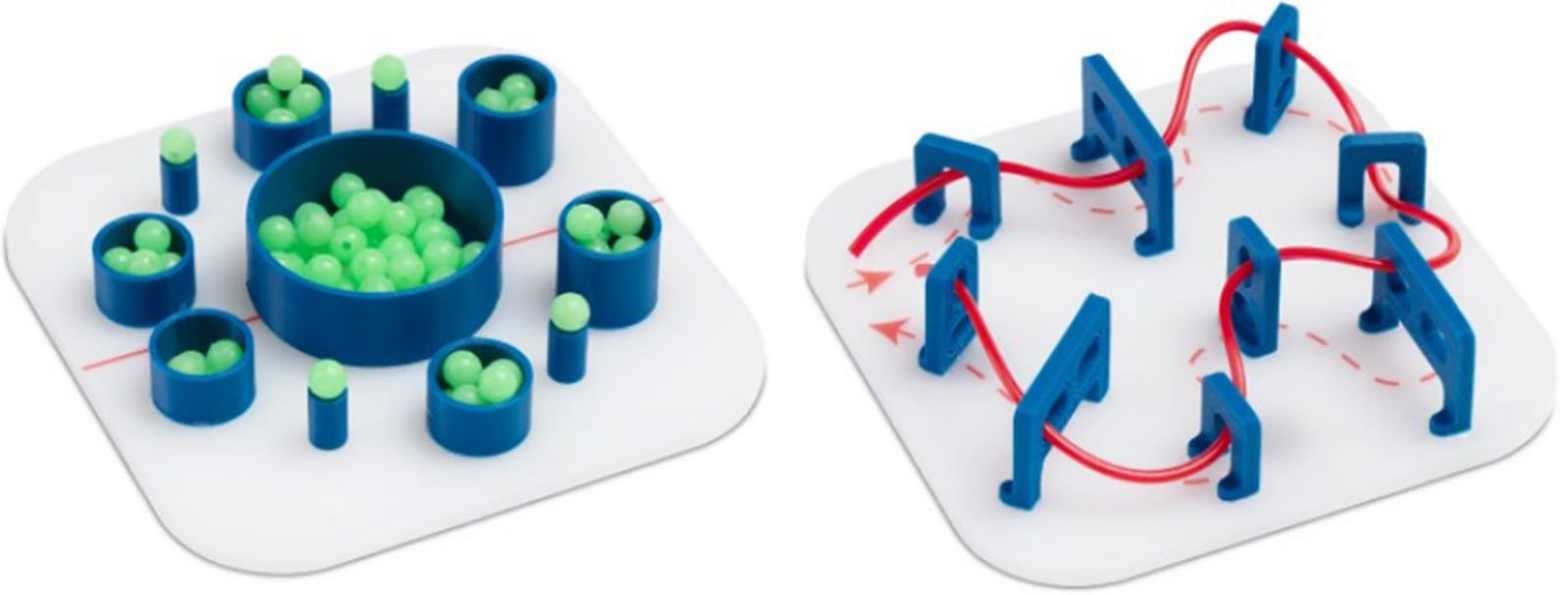
Bead transfer task (left) and threading task (right).

All participants performed the Baseline (Day 1), Post (Day 5) and Retention (Day 10) tests using a laparoscopic skill analytic system. This system allows for detailed capture of a kinematic data from the laparoscopic instruments during completion of the assigned tasks. Participants were assessed on their ability to complete 2 predefined training modules. These were a bead transfer (BEAD) task and a threading task (THREAD). During the bead transfer task, participants were required to transfer 22 beads from a large central pot to 6 small pots and 4 posts by initially grasping the bead with their left instrument, transferring it to their right instrument and then placing the bead appropriately. Participants were asked to complete the task in a clockwise fashion. During the threading task, participants were required to thread a rubber thread through a predefined course consisting of 9 pegs (Fig. 4). During both tasks, participants were instructed to complete the task as quickly, accurately and smoothly as possible, trying to avoid contact with the frame of either task.

### Neurostimulation groups

Each participant was randomly assigned to either an active (n = 26) or sham (n = 23) tDCS stimulation group for the duration of the study at the time of their enrolment. All participants were blinded to the condition they were assigned to, as was the experimenter responsible for administering the testing and training sessions. Participants in both neurostimulation groups wore a custom headset (HALO Neuroscience™) designed to deliver transcranial Direct Current Stimulation (tDCS) to the cortical neurons of the M1 area responsible for controlling hand and arm movement^17^. Nasion, inion and aural landmarks were used to locate the vertex (Cz) on every participant to ensure consistent headset positioning^41,42^. The three studded foam electrodes (24 cm2/electrode) used with the headset were moistened with normal saline (0.9% NaCl) prior to placing the headset unit on each participant’s head to improve conductivity.

Participants in the active (STIM) neurostimulation group received 2.1 mA of bihemispheric stimulation (anode over left M1 and cathode over right M1) for a duration of 20 min whereas participants in the sham (SHAM) neurostimulation group received current that ramped from 0 to 1 mA and then back to 0 mA over two 30 s intervals before remaining at 0 mA for the following 19 min. This sham stimulation protocol has been demonstrated previously to not affect the long-term excitability of the underlying cortical neurons^43^. Participants in both groups completed a crossword puzzle task to control the cognitive engagement of participants and mitigate differences in underlying cortical network activity between neurostimulation groups during the 20-min SHAM and STIM protocols immediately prior to training^44,45^.

### Protocol

**Figure 5:**
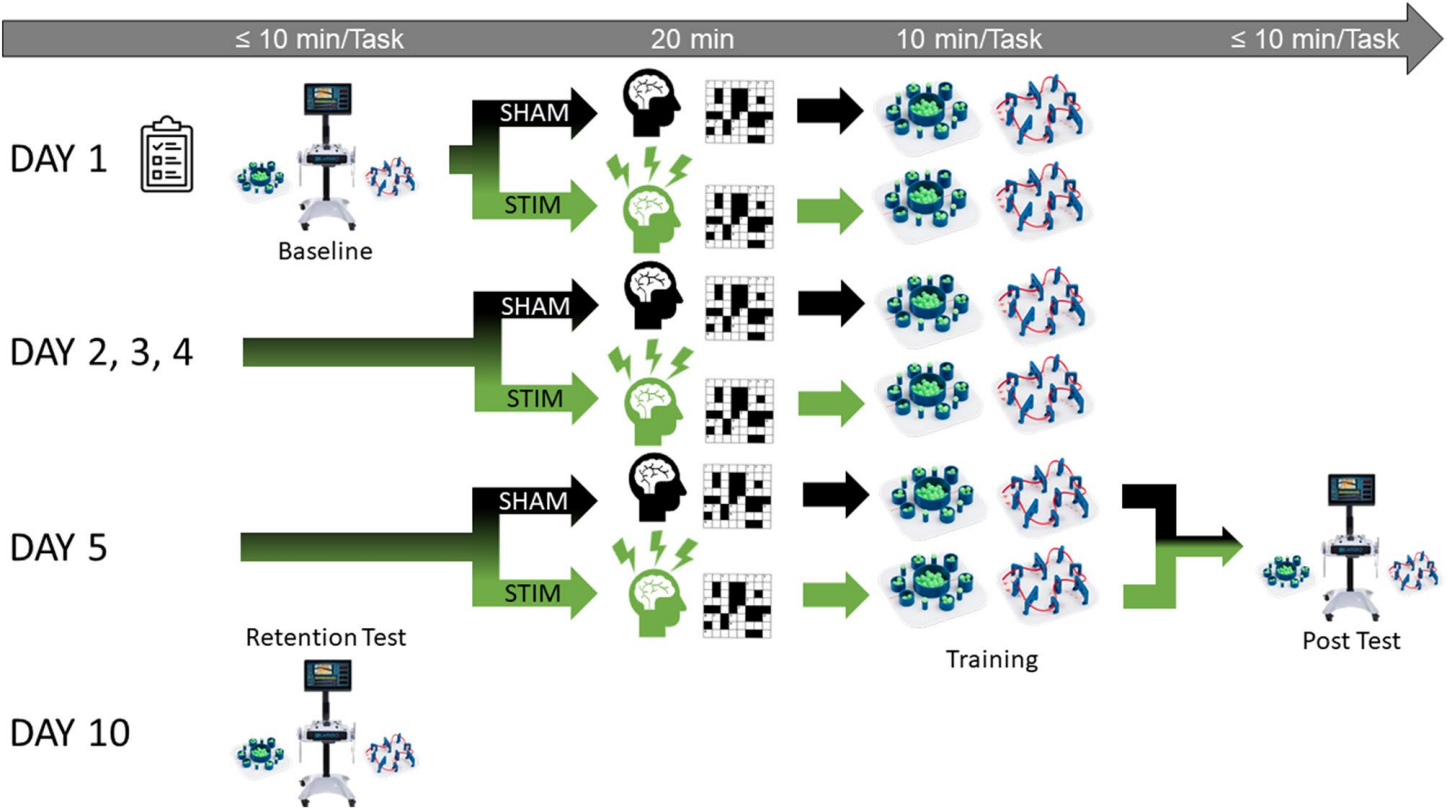
Illustration of the experimental protocol. Both bead transfer and threading tasks were completed for a maximum of 10 min each one after the other at Baseline, Post and Retention test evaluations on the laparoscopic skill analyser.

On the first day (Day 1) participants completed their questionnaires, performed their baseline evaluation of the bead transfer and threading tasks on the laparoscopic skill analyser, completed their neurostimulation protocol, and performed 20 min of laparoscopic training in the box trainer. During test and training sessions, participants were accompanied in the room only by the research team. On days 2, 3 and 4, participants simply completed the neurostimulation protocol and performed 20 min of laparoscopic training in the box trainer. During this training, participants were given standardised instructions by experimenter DG to help them with completing the bead transfer and threading tasks and were asked to complete the bead transfer and threading tasks as many times as they could during the training session. 10 min of bead transfer training always preceded 10 min of threading task training. On Day 5, after completing the BRUMs and PSQI questionnaires a second time, they received the appropriate neurostimulation protocol and trained a final time for 20 min. Following this training session, participants performed their Post evaluation of the bead transfer and threading tasks on the laparoscopic skill analyser. Participants then returned to the lab 5 days later (Day 10) to complete a final Retention evaluation of their bead transfer and threading task performance on the laparoscopic skill analyser and were asked to report which stimulation group they thought they had been assigned (manipulation check) (see Fig. 5 for an illustration of the experimental protocol). During all three testing sessions, the bead transfer task was always performed prior to the threading task. All participants were scheduled for their various test and training sessions at similar times each day to avoid any influence of time of day on test or training performance. Participants who did not complete the Post evaluation or missed 2 training sessions were excluded from the study (3 participants were excluded in this way). Overall, 49 participants were brought forward for data processing and analysis.

### Data processing

Four primary performance metrics were recorded from each of the Baseline, Post and Retention laparoscopic evaluations. The Time to Complete (TTC) each task was determined from the time the instruments were inserted into the laparoscopic box trainer to either the time when they were removed after completing the task or when 10 min had expired. The Distance Travelled metric described the total distance each implement travelled between the time of insertion and either the time of removal or the expiry of the 10-min time limit. The average velocity of each implement was recorded between the start and end of each task as was the number of excessive velocity events, calculated as the number of instances that the instantaneous velocity of an implement exceeded two standard deviations of the average velocity.

### Analyses

All statistical analyses were conducted using SPSS v25 statistical software. Data normality was verified by Shapiro–Wilk analysis and observation of Q–Q plots and histograms. For any metrics where data residuals were not normally distributed, outlying data beyond 1.5 × the inter quartile range were excluded prior to conducting parametric analyses. Independent t-tests were conducted to evaluate where data for any of our metrics differed between males and females. Where Mauchly’s sphericity tests indicated the variance was significantly heterogeneous among groups, Greenhouse–Geisser alpha corrections were applied when conducting ANOVAs and ANCOVAs. Three-way ANOVAs (Session x Condition x Implement) were conducted to test whether performance differed across sessions, between stimulation conditions, or between implements. Where a main effect of condition or interaction between session and condition was found, 3-way ANCOVAs were conducted, controlling for baseline scores, to establish whether a significant effect of session (Post and Retention), stimulation condition, implement, or the interaction between these variables existed.

### Supplementary Information

Supplementary Tables.

## Data Availability

The datasets used and/or analysed during the current study available from the corresponding author on reasonable request.
